# Optimized Protocol for Subcutaneous Implantation of Encapsulated Cells Device and Evaluation of Biocompatibility

**DOI:** 10.3389/fbioe.2021.620967

**Published:** 2021-06-24

**Authors:** Emilie Audouard, Lisa Rousselot, Marc Folcher, Nathalie Cartier, Françoise Piguet

**Affiliations:** ^1^NeuroGenCell, Inserm U 1127, CNRS UMR 7225, ICM, Institut du Cerveau et de la Moelle Épinière, Sorbonne Université, Paris, France; ^2^Department of Biosystems Science and Engineering (D-BSSE), ETH Zürich, Basel, Switzerland; ^3^Institute of Molecular and Clinical Ophthalmology (IOB), Basel, Switzerland

**Keywords:** device implantation, encapsulated cell, biocompatibility, fibrotic capsule, vascularization

## Abstract

Improving a drug delivery system is critical to treat central nervous system disorders. Here we studied an innovative approach based on implantation of a wireless-powered cell-based device in mice. This device, coupling biologic material and electronics, is the first of its kind. The advantage of this technology is its ability to control the secretion of a therapeutic molecule and to switch the classical permanent delivery to activation on demand. In diseases with relapsing-remitting phases such as multiple sclerosis, such activation could be selectively achieved in relapsing phases. However, the safety (tolerance to biomaterials and surgical procedure) of such a clinical device needs to be verified. Therefore, the development of tools to assess the biocompatibility of the system in animal models is an essential step. We present the development of this new therapeutic approach, the challenges we encountered during the different steps of its development (such as cell loading in the chamber, surgery protocol for subcutaneous implantation of the device) and the tools we used to evaluate cell viability and biocompatibility of the device.

## Introduction

One of the main challenges for treating neurological disorders is to develop/improve drug delivery systems to reduce potential adverse effects and thus improve quality of life. The possibility to deliver drugs on demand could be of significant value especially for diseases with alternating disease phases such as relapsing-remitting multiple sclerosis (RRMS). Indeed, RRMS is the most common form of MS and is characterized by periods of relapse (or attacks) and periods of stability with partial or complete recovery of symptoms (Haider et al., 2016). Interferon-beta, the classical medication for RRMS, is administered weekly or even more often, according to the product half-life, via subcutaneous or intramuscular injections. These modes of administration lead to adverse effects such as reactions at injection sites and flu-like symptoms (Madsen, 2017; Saleem et al., 2019). Moreover, repeated administration greatly affects the patient’s quality of life. Therefore, new technologies are needed to improve drug delivery and quality of life and avoid adverse effects.

Two recent technologies, macro-encapsulated cell technology (ECT) and optogenetics, allow for delivery of treatment in a continuous or controlled way, respectively. The ECT therapeutic strategy is based on the confinement of genetically engineered cells within a semi-porous device. The dimensions of the ECT cell chamber are in the order of a centimeter to support the growth of several millions of therapeutic cells. The ECT flat-sheet architecture capitalizes on a cell chamber confined by a semi-porous membrane flanked by a reinforcement mesh (Sweet et al., 2008; Lathuilière et al., 2014). The device contains a lateral filling port for cell loading that is obscured after the loading and before implantation. This design fulfills several device requirements. On one hand, the semi-porous membrane allows the secreted therapeutic proteins to diffuse out of the implant; on the other, the membrane protects the engineered cells against the immunological response of circulating cells (Lathuilière et al., 2015). After subcutaneous implantation, the device allows for diffusion of the secreted protein though the membrane confinement barrier to the vasculature, thus ensuring the biodistribution of the active medicinal ingredient. Furthermore, neovascularization surrounding the cell chamber allows essential nutrients and oxygen to feed the cells via the semi-permeable membrane. One of the main advantages is that in contrast to other cell therapy approaches, ECT is reversible, because the device can be easily explanted. This technology represents an interesting alternative for treating chronic diseases requiring repeated injection of biologics, such as diabetes where the ECT is named GluSense (implantable glucose sensor; patent US2017/00720074A1). Clinical trials are currently ongoing to validate its potential applications for central nervous system (CNS) disorders (NCT01163825^1^) and eye macular degeneration (NCT 01530659 and NCT03071965; see text footnote 1) (Aebischer et al., 1996; Bachoud-Lévi et al., 2000; Bloch et al., 2004; Yizhar et al., 2011; Zhang et al., 2011; Eriksdotter-Jönhagen et al., 2012; Birch et al., 2013, 2016; Lathuilière et al., 2015; Eyjolfsdottir et al., 2016; Lütjohann et al., 2018).

Optogenetics relies on photoactivable proteins to control cellular behavior. The technology is widely used to decipher neural networks but also program drug delivery devices (Ordaz et al., 2017; Kushibiki and Ishihara, 2018; Xu et al., 2020). Optogenetic alleles can be integrated to control excitable cells but also to fine-tune the expression of specific transduction pathways. The wireless-powered optogenetic cell-based device integrates a biophotonic light interface to control recombinant therapeutic protein from the engineered cells residing in the implant cell chamber (Folcher et al., 2014). This optogenetic device has biological properties similar to ECT as well as electronic properties, allowing for control of the secretion of therapeutic molecules. Drug-delivery based on such technology would be of particular interest for RRMS with alternating periods of relapse-remission.

The implantation of medical devices, prosthesis or biomaterial, induces a reaction from the host immune system, known as the foreign body response (FBR). The innate immune system initiates the FBR as a result of the adhesion of proteins and other biomolecules to the surface of the implant/biomaterial/prosthesis, followed by macrophage recruitment and tissue inflammation. Host reactions after implantation have been well studied (Anderson et al., 2008; Klopfleisch and Jung, 2017). Briefly, FBR includes provisional matrix formation, acute inflammation, chronic inflammation, foreign body giant cell formation and fibrosis or fibrous capsule formation. After surgical implantation, the wound healing process is concomitant to the FBR. Wound healing is a well-orchestrated and highly efficient process that can be divided into inflammatory reaction, proliferation and maturation of the newly formed tissue. The healing process engages the interaction of various cell types, soluble cytokines and chemokines (Singer and Clark, 1999). The most notable difference between common wound healing and the FBR is the major influence of the surface of the implanted biomaterial on tissue repair (Klopfleisch and Jung, 2017).

Despite the progress in understanding the general mechanisms sustaining the host response against foreign materials, predicting the response to new biomaterials is challenging. Indeed, the properties of biomaterials play a crucial role in modulating the FBR (it can influence the adhesion, activation and differentiation processes) in the first month after implantation. Moreover, the tissue response at the implantation site might be affected by the implant design and localization, the state of the host area, and the surgical technique. Consequently, the biocompatibility (safety) of the medical device, prosthesis or implanted biomaterial in animal models and humans may be affected by several parameters.

The objective of this work, which falls into the scope of the Optogenerapy European transdisciplinary consortium, was to set up and validate the preclinical testing of a wireless-powered optogenetic cell-based device to treat MS. We aimed to validate the concept and the efficacy of the treatment in the experimental autoimmune encephalomyelitis (EAE) mouse model of MS. Considering that this is an innovative technology before validation of treatment efficacy in the EAE mouse model, we had to define the standard operating procedures, prepare and validate the advanced therapeutic medical device *in vitro* and establish a surgical implantation protocol. We are sharing our experience with this new approach to give advice on the challenges encountered during the experimentation steps. Moreover, as mentioned above, because other parameters affect the biocompatibility of the device and the FBR, we developed tools at each step to assess the biocompatibility in animals.

## Materials

**FIGURE 1:**
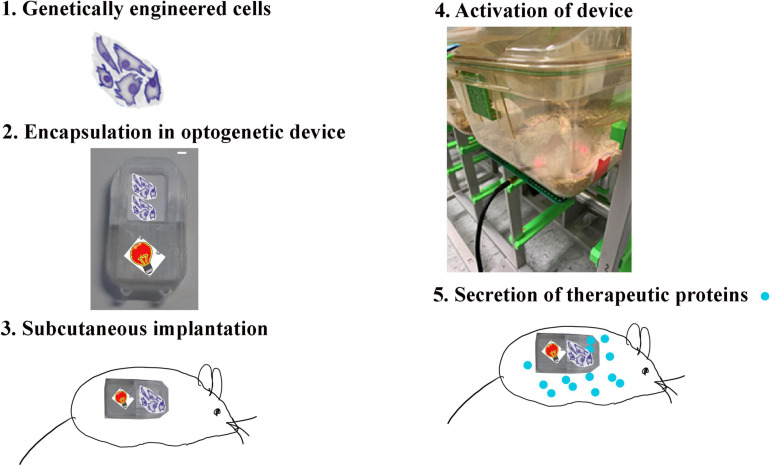
Method of the implantation of the wireless-powered cell-based device. The implantation of the wireless-powered cell-based device [18 × 34 × 3 mm (L × l × H)] in the mouse requires that genetically engineered cells be able to secrete the protein of interest and activate the wireless-powered optogenetic implant. The cells (1) are loaded in the device (2), and the device is implanted in the mouse (3). After complete wound healing, the wireless-powered optogenetic device is activated (4) to secrete the protein of interest in the body of the animal (5).

The implantation of wireless-powered cell-based devices requires genetically engineered cells able to secrete the protein of interest and a wireless-powered optogenetic implant. Cells are loaded in the device before implantation in the rodent. In this context, we must work in aseptic conditions with sterile material at each step to avoid any contamination of the device or animals. After complete wound healing, the wireless-powered optogenetic device is activated in order to secrete the protein of interest in the body of the implanted animals. A radio frequency (RF) antenna is used to activate the implanted device in the animal for several hours. The secretion of the protein of interest is sustained from 2 to 3 days after its activation depending on the cell line and the design of the genetically engineered cells (Figure 1).

### Device

**FIGURE 2:**
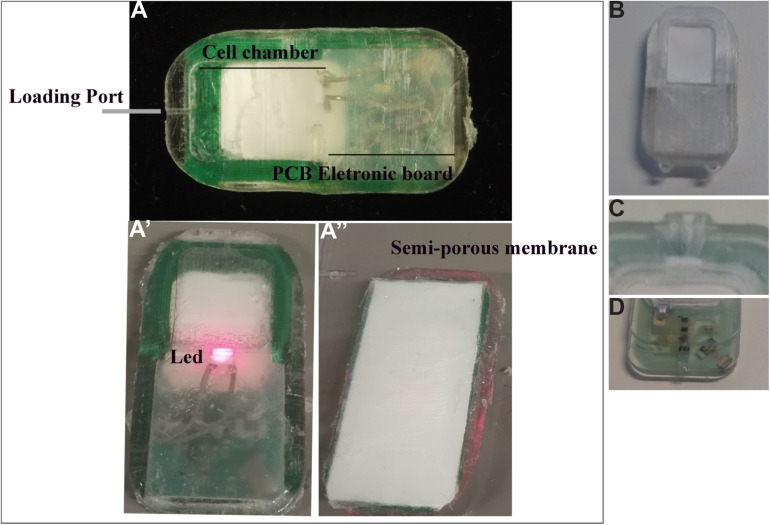
Different types of devices implanted in mice. **(A–A”)** Different compounds of the bioelectronic cell-based device: the loading port allows to load cells in the cell chamber **(A)**; PCB electronic board **(A)** integrates the energy harvesting antenna, the rectifying circuit powering a 680 nm LED **(A’)**. The device cell-chamber membrane confinement barrier is composed of a semi-porous hydrophilic polypropylene membrane **(A”)**. **(B)** 3D-printed implant. **(C,D)** Silicone implants; **(C)** shows the pore to load cells in the implant, and this part creates friction on the skin of the animal, thus promoting lesions; **(D)** indicates the second layer of silicone in which a problem of permeability was observed.

We have used several device prototypes (Figure 2). The final size of the prototype was 18 × 34 × 3 mm (L × l × H), still important in length regarding mouse implantation, but it is the minimal size to have all necessary components for the optogenetic technology. The wireless powered optogenetic cell-based device consisted of a cell chamber containing genetically engineered cells and an optoelectronic part (Figures 2A,B). The bioelectronic cell-based device associates a PCB electronic board integrating the energy harvesting antenna, the rectifying circuit powering a 680 nm LED with a 600 μm thin cell-chamber (Figures 2A,A’). The PCB electronic board is over molded in the implant casing using silicon polymer (Dow silicon 732). The device cell-chamber membrane confinement barrier is composed of a 0.4 μm a semi-porous hydrophilic polypropylene membrane (Pall) covered by a 100 μm rigidifying polyester (RCT Reichel Chemitechnik) mesh (Figure 2A”). The transparent rigid, clear, biocompatible photopolymer (Veroclear Stratasys) casing serves as frame to glue the cell-chamber confinement membranes. The laser-cut membranes were glued to the photopolymerized casing. The gluing assembly process offers a good reliability as it preserves the membrane integrity. The optical window of the cell-chamber enables the *in vivo* visualization of the cells growing in the device. Assembled devices were washed with sterile dH_2_O; followed by repeated 70% Ethanol and dried at 50°c for 1h. Device casing were further exposed to UVB for 1 h in laminar flow cabinet. In contrast, devices manufactured by injection molding (called implant D) were sterilized by ethylene oxide (EO).

### Cell Culture and Genetically Engineered Cells

We used HEK-NirFP (HEK-393 cells stably expressing near-infrared fluorescent protein (NirFP) or hMSC-TERT cells with a synthetic optogenetic pathway controlling a secreted alkaline phosphatase (SEAP) reporter (thereafter called SEAP cells; Folcher et al., 2014). Cells were grown at 37°C in the presence of 5% CO_2_ in DMEM (Gibco 31053-028) supplemented with 10% heat-inactivated fetal bovine serum (FBS; Thermo Fisher Scientific, A3840401), 1% penicillin-streptomycin (10,000 U/ml, Gibco, 15140122) and 1X Glutamax supplement (100X, Gibco 35050-038).

Cell passages need to be monitored carefully. Large batches of 2 million cells/vial were stored in the working biobank at −80°C. Two weeks before implantation, cells were thawed and plated to reach 80–90% confluence on the day of implantation. Moreover, at least 1 day before loading the device, the medium was supplemented with 25 μM biliverdin (Tarutina et al., 2006). Biliverdin, an intermediate component of heme degradation, is used as a chromophore for the phytochrome domain of DGCL and allows the cells to be activated for the optogenetic. Because BV is sensitive to light, these steps should be performed without light and/or by using opaque materials or by covering materials with aluminum paper sheets.

### Mice

All animal studies were performed in accordance with local and national regulations and were reviewed and approved by the relevant institutional animal care and use committee. The experiments were carried out in accordance with the European Community Council directive (2010/63/EU) for the care and use of laboratory animals. Our protocol was approved by the European community council directive (2010/63/EU) (no. 16471).

We used female and male C57BL/6J mice (Jackson Laboratory, cat. no. 000664) between 3 and 6 months old. Mice were housed in a temperature-controlled room and maintained on a 12-h light/dark cycle. Food and water were available *ad libitum*.

### Activation of the Device

After wound healing, the wireless-powered optogenetic cell-based device was activated with the RF antenna (RD5101 HF Long Range RFID Reader) connected to a computer. R-Tool software was used to activate the system. An RF antenna was placed under the cage containing the animal for 4 h to trigger secretion of the protein (Figure 1).

### Equipment Set-Up for the Surgery

The surgery was performed in category A2 animal facilities, and after implantation, animals were housed in A2 animal facilities for the whole procedure.

All surgical instruments and materials are autoclaved in sterile single-use packs. For the surgical procedure, two people are necessary: an assistant (non-sterile) and a surgeon (sterile manipulator). The assistant wears personal protective equipment (white coat, mask, glass, overshoes and long cap) specific to animal facilities. The surgeon wears, in addition to personal protective equipment, sterile gloves and a sterile gown. The surgeon deals only with sterile materials.

In an operating theater, a mobile anesthesia station (Minerve) was installed. This station consists of an isoflurane station, an induction box under a heating blanket, a heating plate with anesthetic mask and multi-station temperature control unit. The system must be turned on to allow time for the blankets to heat up. Moreover, a germinator dry sterilizer (WPI, # 500121) is used to sterilize surgical tools between the surgery for each animal. After surgery, instruments are cleaned (in a 50-ml falcon tube with sterile water) and dried with sterile paper. Devices are placed in germinator and cooled during the surgery.

A sterile surgical field is prepared by covering the operating surfaces (previously cleaned with 70% ethanol) with sterile drapes. The anesthetic induction chamber is set up. The heating pad set to 37°C is placed on the operating surface and covered with a sterile drape. Then the surgical instruments are opened onto the sterile field. The surgeon must wear a sterile gown, gloves, mask, and protective eyewear for the duration of the procedure to maintain sterility of the field. The assistant gives sterile materials to the surgeon.

## Methods

### *In vitro* Validation of Genetically Engineering Cells

Before implantation in animals, a validation of hMSC-TERT cells with a synthetic optogenetic pathway controlling the SEAP reporter was performed *in vitro* to confirm the capacity of modified cells to secrete SEAP in medium as previously demonstrated (Folcher et al., 2014). A total of 150,000 cells/well were seeded on cell culture dishes. The conditions were validated for cells growth in the presence of biliverdin and exposed to near infra-red (NIR) light for 4 h (the optimal illumination time to trigger maximal expression of SEAP). Supernatants for each well (200 μl) were collected before and 24 and 48 h after exposure to NIR light to determine the quantity of SEAP secreted by SEAP cells. SEAP was quantified in culture medium with SEAP Reporter Gene Assay, chemiluminescent (Roche, #11 779 842 001), which confirmed the ability of SEAP cells to secrete SEAP *in vitro* (data not shown).

### Cell Loading Procedure

One must work under strict sterile conditions in a laminar hood to prevent any contamination of the device and inflammatory/immune reaction in the mouse that would compromise the survival of cells and/or the biocompatibility of the implant in the animal. Implants can be prepared the day before or the day of implantation. If the surgery is the next day, we recommend maintaining the filled device in DMEM/F12−/− (i.e., without serum or antibiotics) in a Petri dish until implantation.

Cells are harvested with 0.05% trypsin-EDTA solution (Thermo Fisher Scientific, #25300054) and resuspended in fresh DMEM/F12−/− (Gibco, 31331-028) after cell counting. Cells must be washed twice with DMEM/F12−/− to eliminate all residues of FBS in the device that could interfere with the host body response.

To count cells, 10 μl of the cell suspension is diluted in trypan blue according to the density and count on Kova Slide. According the kind of cell counter, the number of cells per ml is calculated by taking account the dilution factor and/or volume factor.

According to cell counting, a sterile 1.5-ml tube with 2 million cells per tube should be prepared for each implant. After tube centrifugation (1,000 rpm, 3 min, 4°C), cells are resuspended in 200 μl Geltrex 50% (Thermo Fisher Scientific, # 12053569)/DMEM/F12−/−. This step must be performed on ice because Geltrex solidifies at room temperature.

To avoid contamination of sterile devices, sterile gloves and a sterile insulin syringe 0.3 ml (BD, #324826) are used to prevent contamination. A Petri dish 60 × 15 mm is used to store each device before implantation.

**FIGURE 3:**
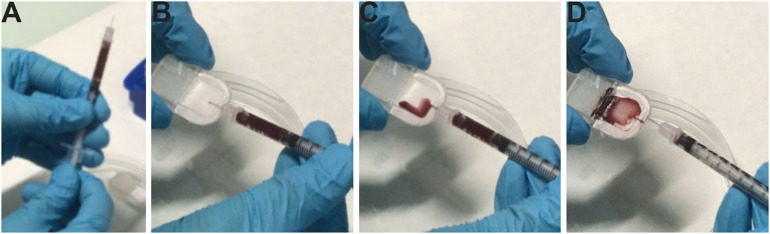
Loading of cells in the device and different troubles encountered. **(A)** Loading of cells in a sterile insulin syringe. **(B)** Insertion of the needle into the pore of the device. **(C,D)** Loading cells in the cell chamber of the device and placing the device in a sterile Petri dish. For more visibility; images were not taken in the culture, and hematoxylin was placed in the syringe.

The insulin syringe is charged with the cells, and the cells are injected in the implant via the loading port (Figures 3A–D). The loading port is sealed with medical glue (Dow Corning 732, multipurpose sealant). The device is placed in a Petri dish and placed in a 37°C, 5% CO_2_ incubator.

### Protocol of Subcutaneous Implantation of the Wireless-Powered Optogenetic Cell-Based Device

#### Assistant

At 30 min before implantation, anesthesia and analgesia (Metacam, subcutaneous 5 mg/kg, Boehringer Ingelheim) is administered to animals to reduce pain due to the procedure. Also, saline solution (0.9%, Braun, 10 μl/g) is injected subcutaneously in the hind limb to avoid dehydration. Animals are shaved. Preventing contamination of the surgery room with the mouse fur is essential, so ideally the mice must be shaved the day before the surgery. Moreover, this step is crucial for the study of device biocompatibility in animals to avoid fur intrusion in the wound that could increase the subcutaneous inflammatory response.

**FIGURE 4:**
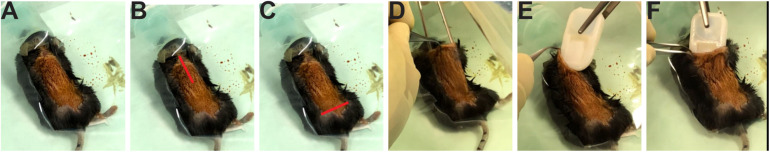
Subcutaneous implantation of the device in the mouse. **(A)** The shaved and disinfected animal is placed on the sterile surface under the anesthetic mask. **(B)** An incision is made on the top of the dorsal back. **(C)** Avoid making a vertical incision in the lower back. **(D)** The subcutaneous tissue is spread with blunt-ended forceps to create a pocket for the implant. **(E)** The skin is held with Graefe forceps and the implant with dressing forceps. **(F)** The sterile device is placed in the subcutaneous skin.

At 30 min after analgesia, anesthesia is induced with 4% isoflurane (IsoVet, 1,000 mg/kg, Vetflurane, Virbac) and is maintained (1.5–2% isoflurane) during the whole procedure (<30 min). Anesthetic depth is assessed by testing the response to various stimuli such as a corneal reflex and toe pinch. To avoid hypothermia, anesthetized mice are kept on a heating blanket to maintain body temperature at 37°C. Sterile eye ointment (10 g, Lubrital, Dechra) is applied on the anesthetized animal for protecting and lubricating eyes. The back skin of the mouse is sanitized with chlorhexidine 0.05% (5%, Hibitane; dilution with sterile water) and the animal is placed on a sterile surface under the anesthetic mask (isoflurane 2%) (Figure 4A).

#### Surgeon

A 1-cm incision is created with a scalpel (Swann Morton # 0506) or iris scissor (WPI, #501758) in the upper part of the dorsal back (Figure 4B). A horizontal incision in the lower back should be avoided because animals may more easily access this region than the upper one (Figure 4C). The skin is held with Graefe Iris Forceps (Allgaier Instrument medical, #08-523-005) and the subcutaneous skin of back from muscle is cleared with dressing forceps (WPI, #500363) to create a surgical window/subcutaneous pocket (Figure 4D). The subcutaneous tissue is spread with blunt-ended forceps to create a pocket for the implant. It is preferable not to fix the device on muscle to avoid its movement; if an adequate pocket is made, it will remain stable for several months.

The sterile device is subcutaneously placed with dressing forceps (Figures 4E,F). The subcutaneous skin and incision are sutured by using absorbable suture Vicryl 6.0 (Vetsuture, PGLA, PGLA07CN) and non-absorbable suture silk 5.0 (Vetsuture LENE, LENE1CN), respectively. The skin or subcutaneous skin is held with Graefe Iris Forceps and the suture thread with an extra-delicate needle holder (with scissors) (Allgaier Instrument medical #19-280-115/F) for a non-continuous suture. The suture must be non-continuous for the subcutaneous layer and the skin, and the stitches must not be too tight to avoid inflammation and/or infection of the wound that would slow the wound healing time.

After suture, the assistant disinfects the wound with chlorhexidine (0.05%) and gives an intramuscular injection of NaCl 0.9% solution (10 μl/1g) to compensate for water loss due to the surgery. Then, the animal is maintained on a warm pad at 37°C during the postoperative period. Water and food are freely accessible in the cage. Gel diet boost (Clear H2O) is added in the cage.

Both sexes of mice can be used for this kind of surgery. However, it is important to be careful after surgery; any problem may prevent the wound from healing properly. The animals must be isolated after surgery. Depending on the strain and sex, animals can be kept together and if trouble occurs after surgery, animals can be isolated for the necessary time and combined again afterward.

### Monitoring Post-surgery

To give the best possible care, animals were monitored daily to follow weight and wound healing. Metacam (5 mg/ml, Boehringer Ingelheim) was subcutaneously injected for 3 days after surgery to reduce postoperative pain. The wound was disinfected with 0.05% chlorhexidine for a minimum of 3 days and longer if necessary.

Scoring parameters were established to assess the immediate inflammatory response and device biocompatibility. The score was assigned on a daily basis by using the following rules: (1) 1 point for wound reopening and (2) 1 point for visible inflammatory response; (3) 1 point if the inflammation persists 3 days after surgery; and (4) 1 point for spontaneous scratching behavior. Data are reported on a scale with a maximum score of 8 points (worst condition) after 3 days of observation. If after 1 week, the score is positive, the animal must be removed from the cohort, and other measures taken to ensure the well-being of the animal.

### The Design Experiment

Before validating the therapeutic effectiveness of the device in the mouse model, the first step was to validate the biocompatibility of the device and validate the different tools to study it. Different device prototypes were implanted in mice for assessment (Figure 2). During this preliminary phase, we encountered different problems that raised important points and allowed for validating the biocompatibility tools developed.

Several groups of mice were used (Table 1): a sham-operated group (*n* = 5) and implanted groups with or without cells. To study the waterproofness of the device, four groups of implanted animals were used: (1) Implant D (silicon prototype) without cells (*n* = 8); (2) Implant D with cells (*n* = 11); (3) Implant A (3D printing device) without cells (*n* = 11); (4) Implant A with cells (*n* = 7). To analyze the EO sterilization of device, two groups of mice were implanted directly (called EO before 1 week, *n* = 10) or at least 1 week after sterilization (EO after 1 week, *n* = 6). Among these different groups, mice were histologically analyzed to validate the different tools of device biocompatibility. We sacrificed mice in at different times: after 7–14 days (EO group with Implant D, *n* = 6), 51 days (*n* = 3; Implant D), 63 days (*n* = 3, Implant A) or 163 days (*n* = 4; Implant D) after implantation.

**TABLE 1:** Different groups of mice.

Sham-operated mice (*n* = 5)	
**Implanted mice**	
To study the waterproofness of the device Implant D = Silicon prototype Implant A = 3D printing device	(1) Implant D without cells (*n* = 8) (2) Implant D with cells (*n* = 11) (3) Implant A without cells (*n* = 11) (4) Implant A with cells (*n* = 7)
To analyze the EO sterilization of the device	(1) EO before 1 week (*n* = 10) (2) EO after 1 week (*n* = 6)
Histological analysis to validate the biocompatibility of device	(1) 7–14 days after implantation—EO before 1 week (*n* = 6)—Implant D (2) 51 days after implantation—Implant D (*n* = 3) (3) 63 days after implantation—Implant A (*n* = 3) (4) 163 days after implantation—Implant D (*n* = 4)

*EO, ethylene oxide.*

### Activation of the Wireless-Powered Optogenetic Cell-Based Device in Animals

Once wound healing is achieved, the wireless-powered optogenetic cell-based device loaded with SEAP cells can be activated. An RF antenna is placed under the cage containing animals for 4 h to activate the optogenetic pathway allowing SEAP secretion in mice blood.

### Blood Samples

Blood sampling is performed under isoflurane anesthesia. Blood is collected from the mandibular vein with a syringe equipped with needle 19G. About 100 μl total blood is collected in a microcontainer tube (BD, #365964A). At 1 h after collection, the tube is centrifuged at 10,000 rpm for 5 min at room temperature. The serum is collected and frozen before analysis in a 1.5-ml reaction tube. Hematology is performed at 4 and 7 days after implantation. The blood analysis gives an estimated count of lymphocytes, granulocytes, monocytes and white blood cells in implanted mice (*n* = 8) vs. non-implanted mice (*n* = 2). Data are collected to detect possible postoperative infection and the inflammatory process. To help the mouse recover from close and repeated blood sampling, NaCl is injected subcutaneously or intramuscularly in animals to compensate for the blood loss.

### Animal Euthanasia

Animals are sacrificed by an intraperitoneal administration of a lethal dose of Euthasol (180 mg/kg, Vetcare) at 7, 14, 51, 63, or 163 days. Mice are shaved, and the tissue surrounding the implant site is collected for analysis. The skin is placed in a cassette (SIMPORT, M505-2) between 2 pieces of foam (SIMPORT, M476-1), then fixed with PFA 4% (Sigma Aldrich, 252549) overnight at 4°C. After washing with PBS 1X, samples are dehydrated with 70% ethanol and embedded in paraffin. During the skin collection, the skin must be cut with a scalpel to have a regular/net square or rectangular piece of tissue to facilitate its positioning in paraffin. The sample must be placed in the mold in a perpendicular manner. Samples are cut at 4− or 6-μm thickness with a microtome (RM2245 Leica). Slides are dried in the incubator at 37°C overnight before further analysis.

### Score to Assess the Device Biocompatibility During Explantation

During euthanasia, scoring parameters are essential to assess the immediate inflammatory response and device biocompatibility. Scoring points are attributed by using the following rules: (1) 1 point for wound reopening; (2) 1 point for visible inflammation of the skin; (3) 1 point for light subcutaneous inflammation (the muscle under device); 2 points for medium inflammation or 3 points for severe inflammation; (4) 1 point for the absence of fibrotic capsule (pocket); (5) 1 point for splenomegaly; and (6) scoring for the purpose of sacrifice (0 point at the end of experiment/1 point or 2 points if animals were sacrificed prematurely due to mild or severe inflammation and/or a reopening of the wound or disruption of the skin). The maximum score is 9 points (worst condition) for the macroscopy assessment of animals during the sacrifice.

### Cell Viability

**FIGURE 5:**
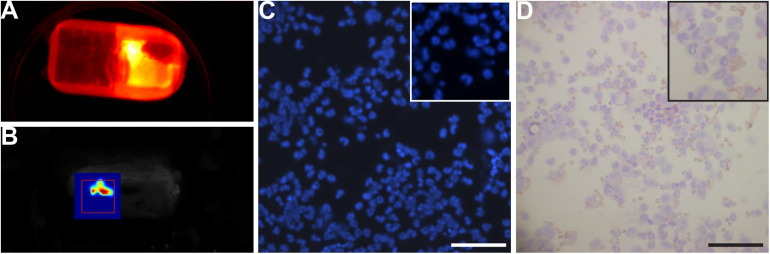
Viability of cells. **(A,B)** Near-infrared fluorescence imaging. **(A)** The device containing HEK-NirFP cells before implantation. Cells were detected. **(B)** The device containing HEK-NirFP cells at 2 weeks after implantation. HEK-NirFP cells were still detectable after 2 weeks of implantation. **(C,D)** DAPI **(C)** and hematoxylin-eosin staining **(D)** of cells in the device collected during device removal. Cells were still present in the device, and the shape/morphology was normal. Scale bar = 200 μm.

Predicting the fate of the cell inside the device during the experiment is difficult. Therefore, the fate must be checked at least at the end of experiment and/or if possible during the experiment. To determine whether cells are still alive/growing during the experiment, the viability of cells after implantation is checked by fluorescent microscopy. An NIR fluorescence imaging system (fDOT) equipped with two lasers (680 and 740 nm) allowing *in vivo* 3D quantitative imaging at resolution mm3 was used for acquisition of images. HEK-NirFP cells were loaded in a 3D-printed device (Implant A without electronics) before implantation. The device loaded with cells and scaffolding material was implanted subcutaneously in mice (*n* = 6). Basal fluorescence emission of HEK-NirFP cells in the device cell-chamber was monitored before implantation with fDOT. A second acquisition was used to monitor the viability of the cell in the implanted device at 2 weeks after implantation. During this acquisition, animals were anesthetized with isoflurane (induction of anesthesia at 4% isoflurane, and a maintenance level 1.5–2%). HEK-NirFP cells were still alive 2 weeks after implantation (Figures 5A,B). Moreover, if we did not have this equipment or complementary to the previous method, during the sacrifice of animals, cells could be collected to analyze the cell viability by trypan blue staining or a kit. Cells were collected from the explanted device with an insulin syringe and placed in a 1.5-ml reaction tube. A sample of cells (about 50 μl) was diluted (1:4) in sterile PBS 1X and placed in a cytofunnel (EZ Cytofunnel Shandon cytofunnel, Thermofisher, A78710003) to shoot out the cells on the Shandon single cytoslide (Thermo Fisher Scientific, 5991059). After drying, cells were fixed with PFA 4% for 15 min. After several washes in PBS 1X, cells were stained with DAPI and/or hematoxylin-eosin to visualize the shape of cells (Figures 5C,D). Another sample of cells (about 10 μl) was used to assess the viability of cells by using trypan blue.

### Assessment of Device Biocompatibility by Histological Analysis

#### Histological Staining

Histological sections were stained with Mayer’s hematoxylin (HE, C0303, DiaPath) and Masson’s trichrome, using standard procedures to visualize the tissue morphology. Vascularization was analyzed by immunostaining with antibody to von Willebrand factor (VWF, directed against blood vessels; Abcam, ab11713, 1:250). Immunohistochemical labeling was performed with the ABC method. Mast cells are visualized by classical toluidine staining (i.e., toluidine 0.1% for 3 min). Images are acquired at 20X by using a slide scanner (Axioscan, Zeiss). Cellular infiltration was assessed with DAPI. Stained nuclei are visualized by using an epifluorescence microscope (Axioscope 2 plus, Zeiss), and images are captured by use of a camera (Axiocam 202 mono, Zeiss).

#### Quantification of Device Biocompatibility

**FIGURE 6:**
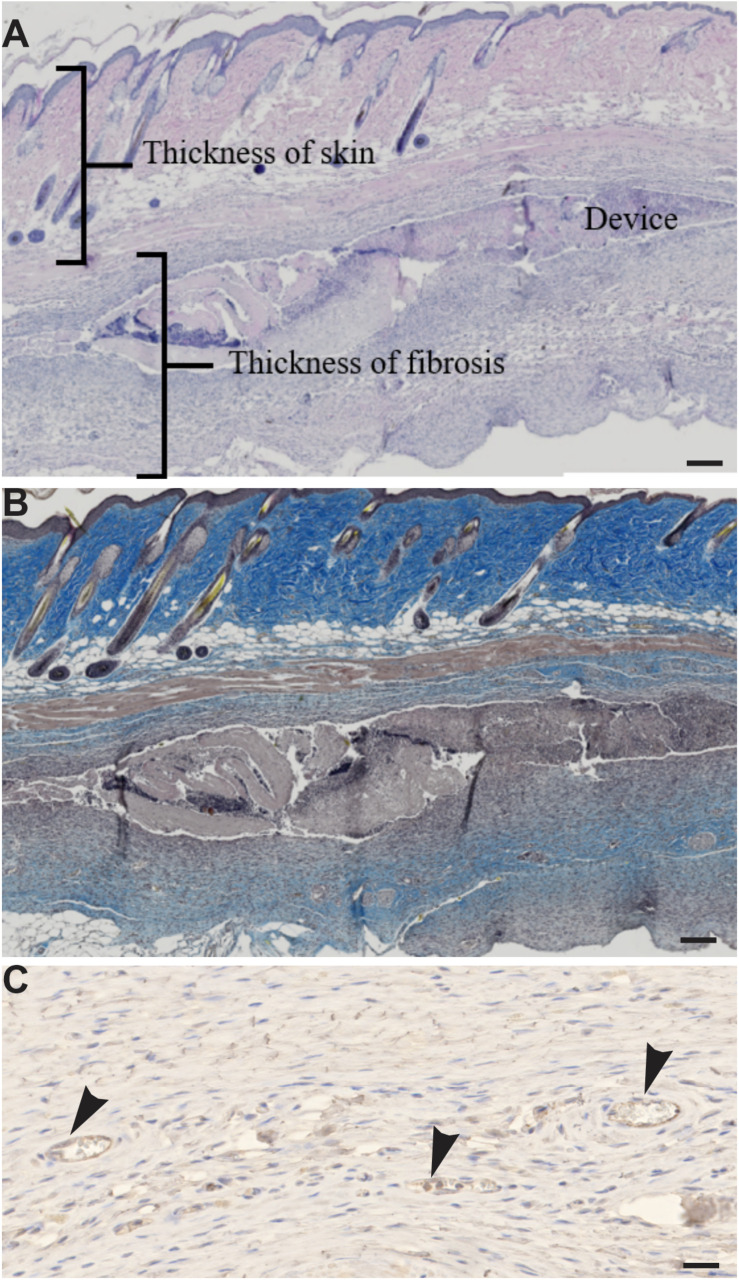
**(A,B)** Tools to assess the biocompatibility of the device. **(A)** Hematoxylin-eosin staining on skin sections. **(B)** Masson’s trichrome staining on skin sections. **(C)** Immunohistochemistry of anti-von Willebrand factor antibody in skin sections shows endothelial cells (arrowhead). Scale bar = 200 μm.

Several parameters were set up to evaluate the biocompatibility of devices in histological analysis. First, the thickness of skin, that is, the thickness between the epidermis and the muscle and the thickness of the dimension at the proximal and distal site of the device, are measured in 5–6 areas of a skin section for each animal (Figures 6A,B). The mean value was considered for each animal.

The area of adipose tissue is measured in a minimum of 50 areas for each animal; the mean value is considered for each animal.

The neovascularization of the device is evaluated by HE staining and confirmed by VWF staining (Figure 6C). The number of blood vessels is counted at the place of the device in 6 areas of the skin section for each animal; a mean by area is calculated for each animal. The diameter of blood vessels is measured in 6 areas of the skin section for each animal; a distribution of blood vessel size is calculated for each animal.

The muscle degeneration is assessed in 6 areas of the skin section for each animal. The number of muscle fibers with a nucleus in the center of fiber is counted for each area; a mean by area is calculated for each animal.

The number of mast cells is assessed by toluidine staining on 5 areas of the skin section (in the region of the device) for each animal; a mean is calculated for each animal.

Cellular infiltration in the region of the device is evaluated by DAPI staining. Five areas of the skin section (in the region of the device) are analyzed; a mean is calculated for each animal. Images are analyzed and quantified by using ZEN software.

### Statistical Analysis

Data were analyzed using GraphPad Prism 8 software. The statistical significance of values among groups was evaluated by ANOVA, followed by the least significant difference *t*-test. All values used in figures and text are expressed as mean ± standard error of the mean (SEM). Differences were considered significant at *p* < 0.05.

## Results

### Advices on the Challenges Encountered During the Experimentation Steps and to Design *in vivo* Experiments

Several problems were encountered during the development of this technology and all the tips/remarks considered as important to point out are reported here, if you want to design such an experiment.

#### General Advices for the Design of This Kind of Technology

##### Shape and size of the device

The design/shape and the size of the device could affect the formation of the lesion, wound healing and implantation in animals (Anderson et al., 2008; Klopfleisch and Jung, 2017). Capitalizing on the experience gained with implantation of different prototypes, we have found that the device must not be too flexible and rough, making it more difficult to implant subcutaneously. From these observations, the device should be (1) small in width, (2) made of smooth material with an ergonomic shape and (3) have well-rounded angles to facilitate its implantation (Figure 2B). It should not have different layers or rigid parts that can create friction or hinder the animal, thus promoting reopening of the wound or lesions (Figures 2C,D). The size of the animal model should be appropriate to the size, shape and material of the device, to avoid forcing during surgery and causing lesions or tears. Here, the weight of animals must be > 20 g to ensure good tolerance of the device.

##### In vitro validation of genetically engineering cells

Before *in vivo* experiments, several parameters must be ascertained: (1) confirm the capacity of cells to secrete the protein of interest; (2) the minimum illumination time of cells to have maximum secretion of the protein of interest and (3) how long this secretion persists to know the frequency of activation needed in animals. These parameters depend on the cell type to be used as well as the secreted protein (efficiency of secretion and half-life).

##### Validation of the cell chamber proprieties

In the design of the project, care should be taken regarding the properties of the cell chamber. (1) The capacity/size of cell chamber must be adapted for the number of cells that need to be seeded in the device to produce an efficient amount of the therapeutic protein and to allow cells to grow several months. This part relies mainly on the choice of cells to be used. (2) The pore diameter of the semi-porous membranes of cell chamber must be selected to (a) allow diffusion of secreted therapeutic molecules out of the implant, (b) allow cells to obtain essential nutrients and oxygen to support growth and (c) protect the engineered cells against the immunological response.

##### Validation of the activation system of device

Important parameters must be preliminarily checked before this first experiment: (1) the power of antenna must be adapted to allow activation of several animals in the same cage; and (2) the emission height of antenna must be sufficient to activate the device when the animal is standing on two legs or when it climbs on the cage grid (but not too powerful to avoid activation of other cages in the vicinity). Therefore, emission of the antenna must be tested by reproducing as much as possible the reality of the situation, taking into account the thickness of the cage and the litter, the body of the mouse and the height of the mouse rearing.

#### Troubles Encountered During the Experimentation Steps

##### Troubles encountered during cell loading

**FIGURE 7:**
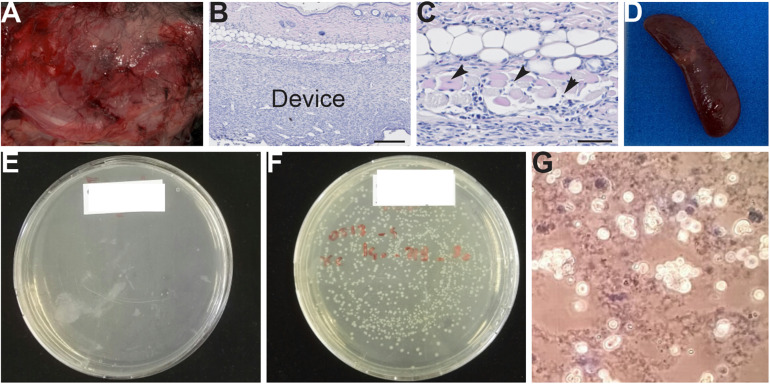
**(A–G)** Macroscopy and histological analysis of animals following problems linked to the waterproofness of the device. **(A)** Severe inflammation observed in tissue (muscle of the back) in contact with the device. **(B,C)** Hematoxylin-eosin staining of skin section. Muscular degeneration is observed, indicating tissue damage (arrowhead). **(D)** Splenomegaly observed in animals showing an infection. **(E–G)** Tests for the confinement of cell chambers/device. **(E)** No leakage was observed with this cell chamber. **(F)** Bacteria were detected in plates, indicating poor waterproofing of the cell chamber. **(G)** The presence of cells and scaffold in a Petri dish, indicating incorrect waterproofing of the cell chamber.

The sterility of cell loading and the confinement of the device are crucial parameters to ensure acceptable tolerance in animals. If the needle does not perfectly fit the injection port, backflow may be observed during the cell loading. The needle gauge is thus an important parameter to ensure aseptic loading and the biocompatibility of the device. Indeed, backflow may contaminate the implant with cells and therefore the animal after its implantation. Moreover, the waterproofness of the device/cell chamber must be perfect. A leakage of cells may be responsible for severe inflammation of adjacent tissue in contact with the device (Figures 7A–C) and more seriously lead to the development of tumors in animals. Indeed, splenomegaly was observed in some animals (Figure 7D), which indicates macrophagic hyperactivity due to infection. To test the waterproof properties of the device, a possible test consists of injecting a bacterial population with a known titer in the cell chamber. Here, the loading volume is set to 350 μl. This volume was chosen to enable the collection of a flow-through material (cell-chamber volume 200 μl). If the implant sealing is correct, the cell chamber should play the role of a sterile filter retaining the bacteria trapped into it. The titer of the bacteria in the flow-through can be enumerated by plating the bacteria on a Petri dish. If the nutrient agar remains clean, then the integrity of the semi-porous membrane is good (Figure 7E). However, if bacteria are growing on nutrient agar, then the membrane is damaged (Figures 7F,G).

##### Desorption of EO residues is a critical step

**FIGURE 8:**
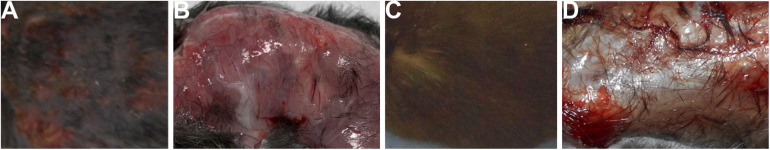
**(A,B)** Severe inflammation on the skin **(A)** and muscle **(B)** in contact with the device after direct implantation and after sterilization with ethylene oxide. **(C,D)** Tissues in contact with the device; implantation was at least 1 week after EO sterilization.

A sterile surgery is a fundamental point to avoid infections and to ensure the tolerance of the implanted device in animals. Thus, the devices must be sterile. In our protocol, devices are sterilized with EO (to be in a clinical condition) or with ethanol then dried in the oven and UV exposed. Regarding EO sterilization, a desorption phase of 1 week is crucial before implantation to allow for extraction of EO residues from the device. Indeed, we observed that when the device was implanted directly or a couple of days after sterilization, severe inflammation was observed on tissues in contact with the device (Figures 8A,B). This phenomenon was not observed when the device was implanted at least 1 week after sterilization (Figures 8C,D).

### Validation of the Biocompatibility of Device *in vivo*

To assess the *in vivo* biocompatibility of the device, we developed a set of tools to determine a “welfare” score for animals post-surgery and at sacrifice, including a blood test and histological analysis of skin sections to evaluate several parameters such the thickness of fibrosis and inflammation due to the device.

#### Follow-Up of the Animal as a Key Step

**FIGURE 9:**
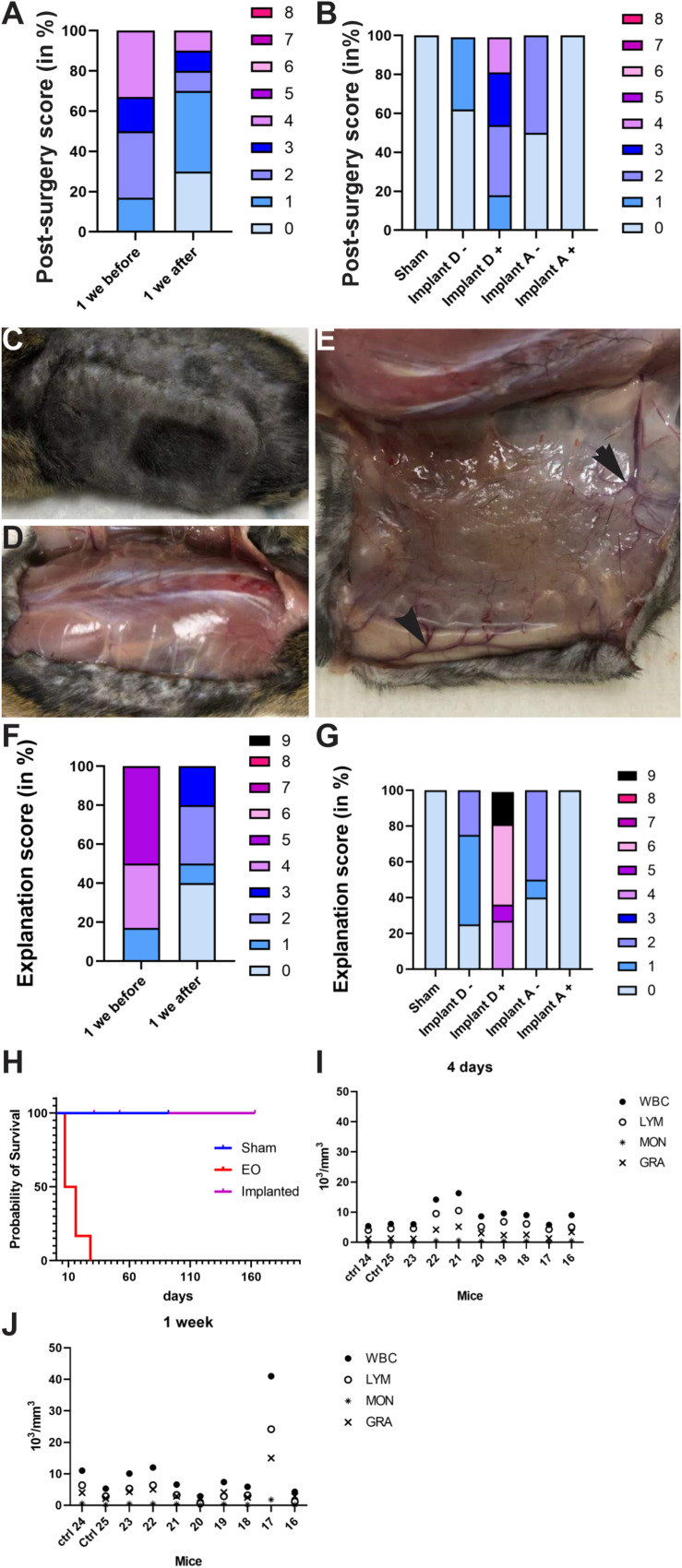
**(A,B)** Histogram of post-surgery score in groups of mice: **(A)** mice directly implanted after sterilization (1 week before) or implanted at least 1 week after sterilization (1 week after) and **(B)** Sham, Implant D loaded or not with cells and Implant A loaded or not with cells. The post-surgery score was high (poor) in mice implanted with device EO and implant D loaded with cells vs. other groups. **(C–E)** Pictures taken during the device removal. No inflammation was observed on the tissue in contact with the device **(C,D)**. **(E)** Neovascularization observed on tissue in contact with the device. **(F,G)** Histogram showing explantation score in groups of mice: **(F)** directly implanted after sterilization (1 week before) or implanted at least 1 week after sterilization (1 week after) and **(G)** Sham, Implant D loaded or not with cells and Implant A loaded or not with cells. Explantation score was very high (poor) in mice with device EO and implant D loaded with cells vs. other groups. **(H)** Survival curves for sham and mice implanted directly after sterilization (EO) or at least 1 week after sterilization (Implanted). 50% of EO animals were sacrificed 7 days after implantation. **(I,J)** Hematology of blood sample in sham and implanted mice 4 days **(I)** and 1 week after implantation **(J)**. No infection was observed in implanted mice in the time exception of the mouse no. 17. WBC, white blood cell; LYM, lymphocytes; MON, monocytes; GRA, granulocytes.

Animals were followed up daily during the first week after implantation to give them the best possible care. To help with/facilitate this follow-up, a post-surgery score was developed. If after a week, the score was not zero, then inflammation or scratching and/or severe reopening of the wound was still present and the animal was sacrificed for its well-being. This situation indicated problems in the biocompatibility of the device. After each implantation, a score in post-surgery was assigned for each mouse during the first 3 days and 1 week after implantation. Implanted mice directly after sterilization in EO (Figure 9A) and the group with Implant D with cells (for which cell leakage was observed, Figure 9B) showed a high score (poor condition) post-surgery as compared with other groups. As previously mentioned in these two groups, severe inflammation was observed from the day after surgery. This observation confirms/illustrates that the first week is the most critical period for problems of biocompatibility of the implant. Therefore, an initial evaluation of the device’s biocompatibility was developed for the best welfare of the animal.

Also, another score was attributed at euthanasia based on the macroscopic observations such as (1) the cause of sacrifice (i.e., at the end of the experiment or owing to a problem preventing the welfare of animals); (2) the presence or not of inflammation on the skin or/and tissue in contact with the implant; (3) the existence or not of a pocket (connective tissue around the device); (4) the presence of splenomegaly, and (5) the healing of the wound or not. If the animal tolerated the device, tissues in contact with the device were healthy, and neovascularization was observed on the fibrotic capsule present around the device (Figures 9C–E). However, if the device was not biocompatible, severe inflammation of tissue in contact with the device was observed (Figures 7A, 8A,B). Similar to the previous score, we observed mostly severe inflammation, lack of healing of the wound and a premature sacrifice of animals in the EO (i.e., 1 week before, Figure 9F) or leakage groups (i.e., implant D with cells, Figure 9G) as compared with other groups. Indeed, the score was 4–9 for the leakage group and 1–5 for the EO group but was 0–3 for other groups. This observation confirms that these two scoring systems for post-surgery and during device explantation are excellent indicators to assess biocompatibility for the best welfare of the animal. Moreover, 50% of animals were sacrificed 7 days after implantation (Figure 9H). These data seem to indicate a problem of biocompatibility in mice during these two experiments of implantation.

Finally, analysis of hematology in animals at 4 days and 1 week after implantation revealed no inflammatory response or infection due to the implantation. Indeed, the number of lymphocytes, granulocytes and monocytes in the blood of implanted animals was similar to that in sham mice at 4 and 7 days after implantation (Figures 9I,J). Moreover, the values obtained for the same mouse were comparable at 4 and 7 days after implantation, which indicates no inflammation/infection present in implanted mice, except for mouse 17. Blood samples are a complementary tool to assess possible infection in animal. The limitation of this tool is the frequency of blood samples taken, which is very limited in mice.

#### Histological Analysis of Device Biocompatibility

**FIGURE 10:**
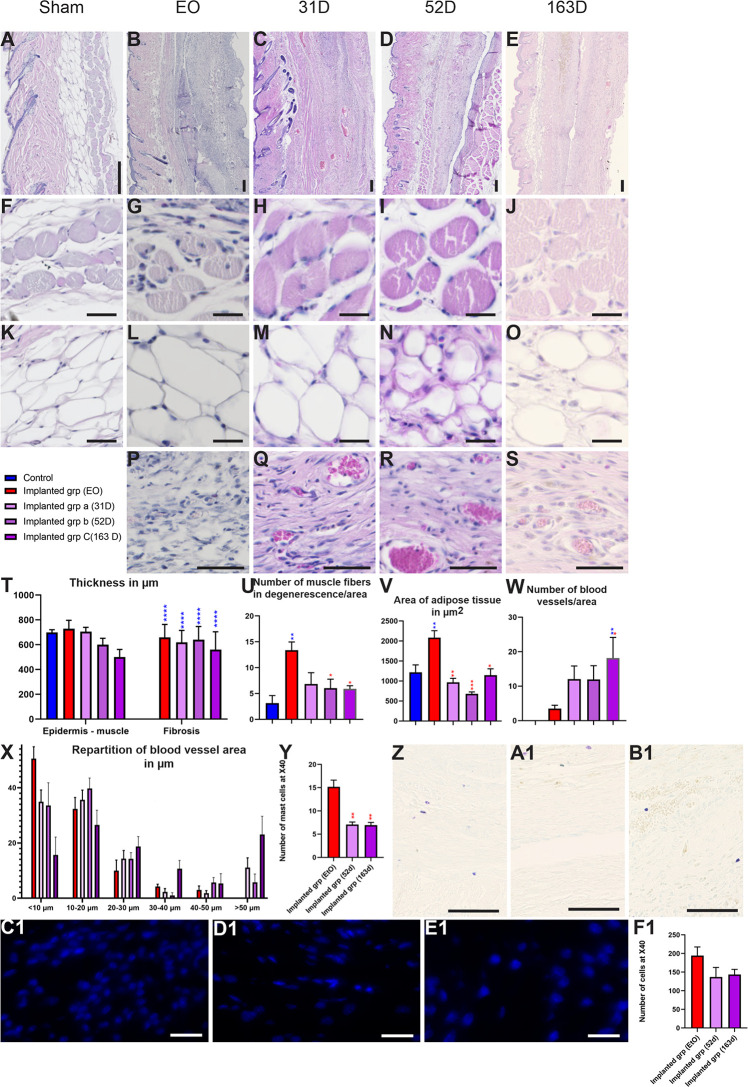
Biocompatibility of devices in implant groups. **(A–S)** Hematoxylin-eosin staining on skin sections in sham **(A,F,K)**, EO **(B,G,L,P)**, 31 days **(C,H,M,Q)**, 52 days **(D,I,N,R)** and 163 days **(E,J,O,S)** groups. **(F–J)** Magnification of muscular area of skin in groups. **(K–O)** Magnification of adipose tissue of skin section in different groups. **(P–S)** Magnification of the neovascularization of the device in EO **(P)**, 31 days **(Q)**, 52 days **(R)** and 163 days **(S)** implant groups **(T)** Quantification of thickness between epidermis and muscle and the fibrotic capsule in sham and implanted groups. **(U)** Muscle fiber degeneration by area. A significant increase in number of muscle fibers in degeneration was observed in EO group in compared to implanted groups. **(V)** Mean area of adipose tissue in groups. EO group showed a significant increase in adipose tissue area as compared with the implant groups. **(W,X)** Quantification of angiogenesis after implantation. **(Y)** Quantification of mast cell density index. Implant groups showed a significant decrease in the number of mast cells as compared with the EO group. **(Z,B1)** Toluidine blue sections show mast cells in EO **(Z)**, 52 days **(A1)** and 163 days **(B1)** groups. **(C1–F1)** Representative DAPI-stained section of the implant demonstrating cellularity in EO **(C1)**, 52 days **(D1)** and 163 days **(E1)** groups. **(F1)** Quantification of DAPI-stained sections. Groups show no significant difference. Scale bar = 200 μm. One-way ANOVA and Turkey’s test *p < 0.05; **p < 0.01; ***p < 0.001 and ****p < 0.0001.

Histological analysis was performed on a skin section in contact with the device (Figures 10A–E). After hematoxylin-eosin and Masson’s trichrome staining of skin sections, we measured different parameters to evaluate the biocompatibility of devices such as the number and size of blood vessels around the device and the thickness of the skin or pocket (Figure 6). The EO group showed a significant increase of muscular degeneration and a significant dilation of adipose tissue, a sign of tissue damage in these animals (Figures 10G,U,L,V). Opposite to the other implanted groups, no sign of tissue damage was observed, and values were similar to the sham group (Figures 10F,K). Moreover, histological analysis of toluidine blue-stained sections showed a significant increase in mast cell numbers in the EO group vs. the implanted group, a sign of acute inflammatory response to implanted biomaterials (Figures 10Z,B1). Finally, DAPI staining of implants and surrounding tissue revealed increased cellular infiltration in the EO vs. implanted group (Figures 10C1–E1). All these data indicate that implants of the EO group were not tolerated by animals. Consequently, these animals were sacrificed before the end of the experiment to respect the welfare of animals. Finally, at least a week after EO sterilization is needed before using implants to eliminate all trace of gas, which was not clear at that time. No problem was encountered after the sterilization of the device with ethanol/UV.

Complementary tools can be used to assess the device biocompatibility in depth. We used three sub-groups sacrificed at 31, 53, or 163 days after implantation and for which the implants were well tolerated. Indeed, these animals showed no tissue damage and good parameters of biocompatibility such as vascularization, no inflammation at the histological analysis or the presence of a pocket (Figures 9C–E, 10). The thickness of connective tissue/fibrosis around the device was similar at the 3 times of implantation. Vascularization around the implant is a sign of the biocompatibility of the device. These groups showed an increase in the number of blood vessels by area as compared with the EO group, and the number was greater at 163 than 31 and 53 days. Moreover, the number of blood vessels with large diameter was increased in the implanted groups vs. EO group (1 week after implantation), which indicates a growth of blood vessels according to the time of implantation. The sub-groups did not differ in number of mast cells and cellular infiltration (DAPI-stained sections) (Figures 10Y,F1). To conclude, all of these data seem to indicate that these different tools are reasonable criteria to assess the biocompatibility of the device.

## Discussion

Implantation of ECT or optogenetics devices represents a new innovative approach with broad-spectrum clinical application in patients. Indeed, the use of continuous subcutaneous insulin infusion and continuous glucose monitoring systems have gained wide acceptance in diabetes care. Moreover, many potential applications in the CNS and the eye (Ordaz et al., 2017) have involved implanting ECT or optogenetic devices. Subcutaneous implantation of ECT for passive immunization against amyloid-beta has been found efficacious in mouse models for Alzheimer disease (Lathuilière et al., 2016; Xu et al., 2020) to reduce brain amyloid and tau pathologies. Optogenetics has allowed for insights into the pathophysiology of many neurological diseases and has also opened doors to therapeutic intervention. The coming years should see the development of new devices directly implantable in the brain. Consequently, this paper is crucial to help in the development of a wireless-powered cell-based device for possible use in MS by giving the detailed experimental procedure and practical solutions to its validation.

The tissue response might be influenced by many other factors such as implant design, implant localization, state of the host bed, surgical technique and mechanical loading (Ibrahim et al., 2017), hence the importance of establishing/developing tools for which we must pay attention to this type of procedure. In this paper, we underline critical points to be considered at each step of the procedure to ensure the success of the validation process or tolerance of the device. Indeed, different parameters in this method are crucial to avoid some postoperative troubles. The first is the ergonomic shape and the size of the device. The shape, ergonomics and size of the device are essential parameters in successful implantation and the welfare of animals (Figure 2). Indeed, these parameters can influence the FBR by stretching the surrounding tissue (Ibrahim et al., 2017). In our case, the prototype with an exterior pore and a double layer of the device led to the reopening of the wound and creating lesions in animals owing to the friction of these parts on the tissue, which reveals the importance of having a smooth and ovoid device. The second critical parameter is the sterility of cell loading, the third is the waterproofness of the device and the fourth is aseptic conditions for the surgery and cell loading. If one of these parameters is not respected, incomplete wound healing or infection will occur. The first week is the most critical to detect a problem with biocompatibility of the device in the animal model. Therefore, the daily follow-up of animals after the implantation is essential during the first week to give the best possible care (Table 2: Summary of different problems that can be encountered in the follow-up of animals).

**TABLE 2:** Troubleshooting.

Trouble	Reason	Solution
Loss of weight	Due to surgery Infection of animal Difficulty to eat/drink	Subcutaneous or intramuscular injection of NaCl Add gel diet in the cage
Reopening the wound	Problem of suture during the surgery or animals to remove it	Suture the wound: Do not make the stitches too tight
Inflammation of the wound	Suture too tight Infection	Disinfection with chlorhexidine or other product
Inflammation of the back	Problem of biocompatibility due to the device: material non-biocompatible or sterilization problem	Disinfection with chlorhexidine or other product If after a week it is still inflamed, sacrifice the animal
Fighting animals	Dominance	Isolate animals
Problem of healing	Animals with grooming Problem of biocompatible device Problem of size device	Isolate animals Find the device problem
Wound other than the incision	Stretching of the skin The morphology of device = friction with skin	Modification of device design

After the surgical implantation of biomaterials, the consequence of the host reaction to the material surface can be devastating for the animal or patient (Anderson et al., 2008). Indeed, we have shown that the presence of EO gas in the implant led to severe inflammation of tissues in contact with the device. EO is known to have potential hazards and toxicity in patients, but it is still a dominant sterilization agent in the medical device industry owing to its effectiveness and compatibility with most materials. The EO adsorption and desorption properties by different polymers used in the medical device industry requires careful verification that EO residues and by-products in medical devices are below hazardous levels before their use in patients (Shintani, 2017). Here, a delay (desorption) of 1 week between sterilization and implantation in animals is needed to avoid any host reaction to EO (Figures 8A,B).

The application of a score post-surgery to determine the immediate inflammatory response and device biocompatibility can help decide the fate of animals (i.e., to sacrifice an animal for its well-being if the animal still presents a high score at 1 week after implantation). Different tools have been developed to assess the biocompatibility of devices in animals (Figures 9A,B). The first is application of a score before the sacrifice of animals to determine the state of tissue surrounding the device and the size of the spleen as a first assessment of the device biocompatibility in animals. The second is histological analysis with several staining’s of tissue (hematoxylin-eosin, Masson’s trichrome and toluidine blue for mast cells) and DAPI staining to assess the cellular infiltration to the implants. The biocompatibility of implanted materials is determined by the intensity of the responses and the ability to resolve injury to the tissues during implantation (Ibrahim et al., 2017). For this reason, histological analysis of tissue surrounding the device is essential to complete this assessment (Figures 6, 10). Finally, hematology can determine the presence of infection (Figures 9I,J). Classically, sections are stained with Masson’s trichrome and/or hematoxylin-eosin to analyze the tissue (i.e., the assessment of FBR (severity of inflammatory reactions), degree of tissue repair and the presence of a fibrotic capsule (Figures 6A,B). Moreover, the neovascularization that develops close to the implanted device is a good way to evaluate biocompatibility (Figure 6C). Indeed, access to oxygen and nutrients is a limiting factor and directly affects the survival and expansion of the cells inside the device. Oxygen and nutrient supply are primarily determined by the neovascularization that develops close to the implanted device (Lathuilière et al., 2014). The presence of a dense network of capillaries near the device/membrane and the proliferation of endothelial cells (an increase in angiogenesis) adjacent to the device/membrane are key parameters essential for optimal survival of encapsulated cells and attest to the device’s biocompatibility (Figure 10).

Further histological analysis may characterize in detail the biocompatibility of the device. The thickness of subcutaneous tissue and/or thickness of tissue expansion/fibrosis and/or analysis of adipose tissue formation are other tools to assess the biocompatibility of biomaterial (Sarkanen et al., 2012; Ibrahim et al., 2017). Some inflammatory cell types such as macrophages, giant cells or mast cells can be quantified by histological staining or immunohistochemistry (Ibrahim et al., 2017). In addition, different scores more or less complicated can be assigned to evaluate the local immunological effects of implantation based on the presence or not of different criteria (collagen, giant cells, lymphocytes, and vascularization etc.) (Ibrahim et al., 2017) or the quantification of inflammatory cell types (Sarkanen et al., 2012).

Finally, an essential question in this kind of experiment is to know whether cells are still alive/growing during the experiment or at least at the end of the experiment. Bioluminescence microscopy or *in vivo* imaging could be used to detect fluorescence signals emitted by cells (if it is available). The advantage of this approach is that one can follow the growth of cells and determine how they develop at the time. We detected cells from the beginning of the experiment and still alive 2 weeks after implantation. Lathuilière et al. (2014) showed that cells were still active at least 19 weeks after implantation. If this kind of equipment is not available, one can collect cells contained in the device during explantation to assess cell viability. We confirmed the presence and state of cells by collecting them after explantation of the device for up to 5 months.

This reported implantation could be extended to all strains of mice and rats. Moreover, all types of cell encapsulation devices, such as encapsulated cell technology (ECT), optogenetic devices, implant membranes or dialysis pumps and all transfected cell lines with a synthetic optogenetic pathway controlling the therapeutic molecule can be used in this protocol.

Once all steps of development and setting up of the implantation process and biocompatibility are achieved, the next steps deal with the assessment of treatment efficacy to established a proof of concept. To assess whether the treatment is efficient, the following experimental groups will be required: non-implanted, implanted ON, implanted OFF, and positive control (i.e., treatment control to verify the efficacy in treated animals). According to the disease and the phenotype of the animals used, one must check the behavioral improvement of animals and/or perform histological or/and biochemical analysis to validate the efficacy of treatment in comparison to untreated animals. Moreover, the bioavailability of the protein of interest in the animal’s blood is an important parameter to check. To determine whether the cells have well-secreted proteins of interest in mice, we could perform different experiments. According to the molecule of interest used or the construction of the genetically engineered cells (e.g., a tag to facilitate its detection in tissues), one can verify *in vivo* its bioavailability in the blood of animals by using an ELISA kit, which would be tested *in vitro* beforehand. However, detecting the molecule with the kit is difficult because it is rapidly used/metabolized in the mouse body, but it can be detected *in vitro* with no problem. Another possibility is to freeze cells in the device directly after explantation.

In our case, the next steps will be to validate this new technology of wireless-powered cell-based device and the efficacy of treatment in the EAE mouse model of MS.

## Conclusion

In conclusion, in this paper, we describe the critical points to be considered when using encapsulated cell device implantation. This innovative approach can be applied to several CNS disorders including Alzheimer disease (Lathuilière et al., 2015, 2016; Lathuilière and Schneider, 2017; Ordaz et al., 2017) and also other devastating pathologies including lysosomal storage disorders such as metachromatic leukodystrophy alone or combined with other therapies to supplement sustained enzyme delivery.

## Data Availability Statement

The raw data supporting the conclusions of this article will be made available by the authors, without undue reservation.

## Ethics Statement

The animal study was reviewed and approved by the 16471.

## Author Contributions

EA, FP, and MF designed the experiments. MF provided HEK-NirFP or hMSC-TERT SEAP cells and 3D-printing devices. EA, MF, and LR performed the experiments (with the help of FP for implantation). EA, MF, FP, and NC wrote the manuscript. All authors contributed to the article and approved the submitted version.

## Conflict of Interest

The authors declare that the research was conducted in the absence of any commercial or financial relationships that could be construed as a potential conflict of interest.

## Footnotes

1 http://clinicaltrials.gov
